# Reactions of Polyfluorobenzenes With Nucleophilic Reagents^1^

**DOI:** 10.6028/jres.067A.050

**Published:** 1963-10-01

**Authors:** Leo A. Wall, Walter J. Pummer, James E. Fearn, Joseph M. Antonucci

## Abstract

Nucleophilic reactions of hexafluorobenzene and related polyfluorobenzenes were studied in detail. Reaction of hexafluorobenzene with hydroxides, alcoholates, aqueous amines, and organolithium compounds led to the substitution of one or more fluorine atoms. The structures of the products were determined, using near infrared and nuclear magnetic resonance spectra. Fluorine is replaced more readily than chlorine, bromine, iodine, or other groups. In the majority of the products in which two of the fluorines in hexafluorobenzene were replaced, the substituting groups were para to each other. However, depending on the reagents other orientation effects were noted. The reaction mechanisms were a function of reagents and conditions. The most prevalent mechanism is presumably the displacement of a fluoride anion by another anion, probably via the formation of transition complexes of different lifetimes. However, simple ionization or attack by neutral species may occur under some conditions. The diazotization and oxidation of pentafluoroaniline were also investigated.

## 1. Introduction

Hexafluorobenzene [1, 2, 3, 4]^2^ and other highly fluorinated benzene derivatives [5] have been synthesized in recent years by several methods. These substances are very inert chemically and extremely stable to heat and radiation [6, 7, 8]. Hexafluorobenzene has not, to date, been reported to react with electrophilic reagents. Exceedingly drastic acidic conditions have been required to halogenate further tetrafluoro- and pentafluoro benzene [9, 10, 11, 12]. Hexafluorobenzene will add chlorine [1, 2, 4] and is readily attacked by free radicals [13].

Like hexachlorobenzene [14], hexafluorobenzene would be expected to react with bases. Strong nucleophilic reagents will displace fluorine from the aromatic ring. The strong inductive effect of the electronegative fluorine atoms should produce highly positive ring-carbon atoms. Unlike hexachlorobenzene, which is a high-melting solid poorly soluble in organic solvents, hexafluorobenzene is a liquid and is soluble in many solvents. Hence, the latter compound readily reacts with many bases in appropriate solvents. Under pressure at elevated temperatures, aqueous systems have also been used successfully with hexafluorobenzene.

The reactions of hexafluorobenzene and many of its monosubstituted derivatives with various nucleophilic reagents will be described in this paper. The chief reagents utilized were alkali-metal hydroxides, alcoholates, amines, and organolithium compounds. In general, these reagents replace fluorine in preference to bromine or iodine, for example, in reactions with bromo- and iodo-pentafluorobenzene. This is in accord with previously observed results on nucleophilic reactions [15, 16]. With 1-chloro-3,4,5,6-tetrafluorobenzene, the chlorine atom can, however, be replaced preferentially by a hydrogen atom [17]. The reaction is carried out with hydrogen over a palladium catalyst at 280 °C.

Many of the reactions studied are informative of the directional effects created by groups other than a fluorine atom in an otherwise completely fluorinated benzene ring. Very meager information of this nature has been available previous to this work. Nuclear magnetic-resonance spectroscopy was used as the primary method for determining molecular structure. Infrared spectra, particularly in the near-infrared, were also of assistance in determining structure, especially in the case of the *ortho* hydroxyl compounds.

## 2. Reactions of Hexafluorobenzene

### 2.1. Reactions With Hydroxides

It has been found that polyfluoroaromatic phenols can be prepared readily by the following type of reaction:

**Figure:**
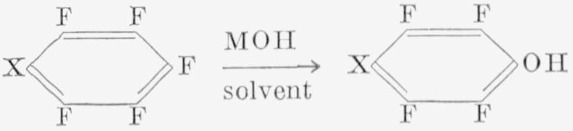

where M is any alkali metal and X is a fluorine atom or some other substituent group. The product is often the *para* isomer, but mixtures of isomers may occur.

In our earlier work [18], pentafluorophenol was prepared by refluxing hexafluorobenzene with potassium hydroxide in pyridine, which was found to be an effective solvent for this reaction. In similar reactions with hexachlorobenzene, it has been suggested [14] that pyridine has a specific, catalytic effect. With ethanolic pyridine as solvent, the reaction gave pentafluorophenol and tetrafluorodihydroxybenzene together with small proportions of pent afluorophenetole and diethoxytetrafluorobenzene. Since the phenol reacts further only with difficulty due presumably to the repulsion of the negative ion by the phenoxy ion, it is likely that the disubstituted products result via reaction of the base with the phenetole and that many substituted ethoxy groups are then split by the basic medium to give the hydroxy derivatives. With *tert*-butyl alcohol as diluent, other workers have prepared pentafluorophenol from hexafluorobenzene [19].

In the present work, it was found that simple, aqueous systems are adequate for these nucleophilic reactions. Thus, we have prepared pentafluorophenol in high yield (85%) by using aqueous potassium hydroxide at 175 °C in a closed pressure-vessel. For the phenol preparation, our experience suggests water to be the preferred diluent, followed by *tert*-butyl alcohol. Tetrafluoro-*p*-cresol was prepared, using *tert*-butyl alcohol as diluent. With pyridine as solvent, black tars are undesired by-products; using pyridine, the compounds 2,3,5,6-tetrafluorophenol, 2-bromo-3,4,5,6-tetrafluorophenol, 4-bromo-2,3,5,6-tetrafluorophenol, and 2,3,5,6-tetrafluoro-4-iodophenol have been prepared.

### 2.2. Reactions With Alkoxides

Another reaction of polyfluoroaromatics investigated was that with such alcoholates as sodium methoxide.

**Figure:**
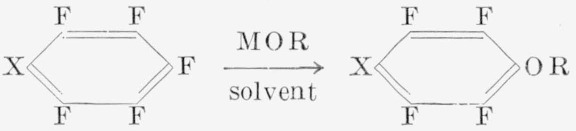

The reaction is usually carried out at reflux temperatures, to give good yields of ethers of the general type shown. With sodium methoxide in methanol, pentafluoroanisole was produced [4, 20], together with some of the tetrafluoro-*p*-dimethoxybenzene. With pyridine—methanol as the solvent [18], rapid reaction occurred to produce high yields (70%) of pentafluoroanisole. The tetrafluoro-*p*-dimethoxy benzene has also been produced by other methods [21, 22], e.g., from the reaction of diazomethane with 2,3,5,6-tetrafluorohydroquinone. Synthesis by this route established that the second methoxyl group is in the *para* position to the first.

The pentafluoroanisole may be demethylated, with difficulty, by refluxing with 47-percent hydriodic acid [3], to give a 20-percent yield of pentafluorophenol. Demethylation is also relatively poor with hydrobromic acid [20]. However, when the pentafluoroanisole is heated with anhydrous aluminum chloride and the mixture is poured onto ice, a 58 percent yield of pentafluorophenol [20] is obtained.

Pentafluorophenol is extremely resistant to further substitution by nucleophilic reagents, probably because these reactions would require the attack of a negative species on the negative phenoxide ion. Ammonium 2,3,4,5,6-pentafluorophenoxide was the only product obtained from treatment of (a) pentafluorophenol, (b) sodium pentafluorophenoxide, or (c) ammonium pentafluorophenoxide with sodamide in liquid ammonia. Thus, the tetrafluorodihydroxybenzene [18] produced in the reaction with ethanolic potassium hydroxide in pyridine probably arises form the attack of a nucleophilic species on the phenetole. In another study [19], only the phenetole was produced in 48 percent yield, and 45 percent of the hexafluorobenzene was recovered after reaction with ethanolic potassium hydroxide for 30 min at 120 °C. In accord with this result is the observation made in this work that the treatment of pentafluoroanisole with aqueous ammonia results in the isolation of ammonium pentafluorophenoxide. With hydrazine hydrate in ethanol, hydrazinium pentafluorophenoxide is obtained [20]. However, benzyl pentafluorophenyl ether is not readily split by nitrogen bases, and a fair yield (13%) of benzyloxytetrafluoroaniline was obtained by treating it with aqueous ammonia.

In this work, bromo- and iodo-pentafluorobenzene were converted to the corresponding anisoles in which a fluorine atom is replaced by a methoxyl group. Reaction of sodium benzyloxide (in excess benzyl alcohol or in *tert*-butyl alcohol) with hexafluorobenzene gave benzyl pentafluorophenyl ether. A higher yield of purer material was obtained when the latter solvent was used, since benzyl alcohol is difficult to remove from the product.

Anhydrous sodium phenoxide was found to react rapidly with hexafluorobenzene at room temperature in *N*,*N*-dimethylformamide, giving 2,3,4,5,6-pentafluorophenyl phenyl ether. This compound was also prepared by treating potassium pentafluorophenoxide with bromobenzene, using activated copper as a catalyst. This reaction failed to give the perfluorophenyl ether when bromopentafluorobenzene was used instead of bromobenzene; the starting materials were recovered.

Perfluorophenyl ether was, however, prepared, although in relatively low yield, by reacting potassium pentafluorophenoxide with hexafluorobenzene in *N*,*N*-dimethylformamide. This reaction was more sluggish than that involved in the preparation of the pentafluorophenyl phenyl ether and required refluxing. This difference may be partially explained by the poorer nucleophilic nature of the potassium pentafluorophenoxide as compared to the sodium phenoxide.

By the reaction of sodium ethoxide in ethanol with pentafluoro-*N*,*N*-dimethylaniline, 4-ethoxy-2,3,5,6-tetrafluoro-*N*,*N*-dimethylaniline was synthesized. No pyridine was used in this reaction, since phenol formation is likely to occur under these conditions.

### 2.3. Reactions With Amines

Hexafluorobenzene has been shown to undergo reaction with sodamide at −70 °C in liquid ammonia, to give 2,3,4,5,6-pentafluoroaniline [23]. In ether, reaction with sodamide did not produce pentafluoroaniline; a solid which slowly sublimed at 85 °C was obtained [24]. The preferred reaction for replacing fluorine atoms with amino groups is treatment with aqueous amines [14].

In this investigation, the reaction of aqueous amines with polyfluoroaromatic compounds has been extensively explored.

**Figure:**
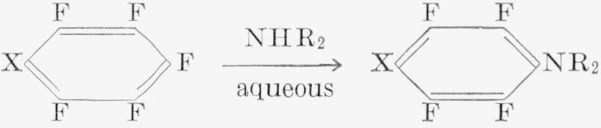

Our result differed in certain respects from those obtained when aqueous ethanolic amine solutions were used [25]. The reactions were carried out at 160 to 230 °C in a steel pressure-vessel. Control of temperature and time of the reaction is critical since, at low temperature, the reaction does not occur and, at higher temperatures, unidentified tars are produced.

In this investigation, bromo- and iodo-tetrafluoroanilines, pentafluoroaniline, and various polyfluoromethylanilines were prepared by the aqueous amine process. Although (benzyloxy)tetrafluoroaniline was formed by the reaction of benzyl pentafluorophenyl ether with aqueous ammonia, only ammonium pentafluorophenoxide was produced from 2,3,4,5,6-pentafluoroanisole. It was found, however, that, in the reaction of pentafluoroanisole with sodamide in liquid ammonia, normal replacement of a fluorine atom occurred (instead of cleavage of the methoxy group) to give 2,3,5,6-tetrafluoroanisidine. Since the fluoroanisidine formed is amphoteric, such products as the di- and tri-anisylamines were also formed.

#### a. Diazotization of Pentafluoroaniline

The versatility of the “diazo reactions,” i.e., the Sandmeyer reactions, is well known in aromatic chemistry as they provide a means of preparing a variety of compounds not obtainable by other methods. It was of interest, therefore, to determine the usefulness of the Sandmeyer reaction for preparing pentafluoroiodobenzene and bromopentafluorobenzene.

Pentafluoroaniline is weakly basic, reacts slowly with nitrous acid, and dissolves only in concentrated strong acids [23]. On dilution with water, however, the free base is regenerated; this demonstrates that the salt of the amine hydrolyzes readily, which is an important factor in the diazotization process in aqueous solutions. Diazotization of pentafluoroaniline in anhydrous hydrogen fluoride and concentrated sulfuric acid has been studied by us. It can be oxidized to give a variety of products; pentafluoronitrobenzene [26], pentafluoronitrosobenzene [27], and decafluoroazoxy benzene are described herein.

The diazotization of pentafluoroaniline to yield decafluorodiazoaminobenzene, $C_{6}F_{5}—N=N—\overset{H}{N}—C_{6}F_{5}$, has been reported previously [23], but no experimental details were given. We have found that this compound is obtained when concentrated hydrobromic acid (48%) is used as the reaction medium; its formation may be attributable to the slow diazotization of the amine (rather than to a too low concentration of acid, which is sometimes the cause for this coupling reaction [28]). The reaction also appears to be temperature-dependent, because the rate of formation of the diazoaminobenzene is much faster at 10 °C than at −10 °C. Allowing the latter reaction mixture to warm to 5 °C causes the compound to be precipitated.

Decafluorodiazoaminobenzene can be decomposed in warm hydrobromic acid solution in the presence of cuprous bromide. The products obtained are bromopentafluorobenzene (5.4%) and a mixture of dibromotetrafluorobenzenes (11.7%), presumably the *o*- and *p*-isomers. The isolation of the latter compounds suggests that the diazo group exerts a considerable influence on the *ortho* and *para* fluorine atoms, making possible the replacement with bromide anion. This replacement reaction apparently occurs prior to the decomposition of the diazo group, because bromopentafluorobenzene is stable to concentrated mineral acids [12, 29]. Similar replacement reactions have been observed [23] when the diazotizations were performed under neutral or alkaline conditions.

In such nonaqueous solvents as concentrated sulfuric acid, the diazotization of pentafluoroaniline is extremely slow, even at 25 °C. The addition of glacial acetic acid tends to hasten the reaction slightly. The deep-red color appears only after 24 hrs. Deamination with hypophosphorous acid yields a product believed to be a mixture of pentafluorobenzene and tetrafluorobenzene. These compounds are difficult to separate, even by vapor-phase chromatography. Dilution of the diazo solution with water reprecipitates the yellow diazoaminobenzene, as mentioned previously.

The use of anhydrous liquid hydrogen fluoride as the solvent and reaction medium resulted in a decided improvement in the yield of product. Liquid hydrogen fluoride has found moderate use in diazonium reactions, even though fluorobenzene can be prepared in 86 percent yield from aniline in this medium [30]. We have found that the diazotization of pentafluoroaniline is fairly rapid, even at −25 °C. Pentafluoroidobenzene and bromopentafluorobenzene can be prepared in 50 percent and 35 percent yield, respectively, by normal Sandmeyer reagents (potassium iodide—or potassium bromide— cuprous bromide mixture). No other replacement products were observed during these reactions.

Another synthesis involving diazotization was the attempted preparation of the fluorinated diazo ether C_6_F_5_—N=N—O—C_6_F_5_. Thermal decomposition of this material could possibly lead to the hitherto unknown perfluorophenyl ether. The product isloted from the reaction of lithium pentafluorophenoxide and the diazotized amine could be extracted into methylene chloride. However, after partial removal of the solvent, the material detonated violently. The instability of some substituted aroyl diazo ethers is well known [28], and our results indicate that this fluorodiazo ether may also he unstable.

The Sandmeyer nitrile synthesis failed to give any identified products with diazotized pentafluoroaniline. It has been previously observed [12, 29] that cyanide ion has an adverse effect on aromatic fluorine compounds, causing multiple replacement with subsequent decomposition, followed by carbonization. These results indicate that the diazonium reaction may have only limited usefulness in aromatic fluorocarbon chemistry.

#### b. Oxidation of Pentafluoroaniline

The oxidation of aromatic amines to nitro derivatives has been accomplished previously by using peroxy acids [31, 32, 33]. This procedure can also be applied to pentafluoroaniline [26, 27], and both pentafluoronitrobenzene [26] and pentafluoronitrosobenzene [27] have been synthesized. For oxidation of pentafluoroaniline, we used the conditions described by Holmes [34]. However, instead of the expected pentafluoronitrosobenzene, only decafluoroazoxybenzene was isolated as the final product, regardless of whether the reaction was allowed to proceed at room temperature or if heat was applied; when heat (60 to 75 °C) was used, the reaction proceeded mush faster. Presumably, from the color changes observed during the course of the reaction, it should be feasible to isolate the pentafluoronitrosobenzene by quenching the reaction at the green-color stage. Pentafluoronitrosobenzene has been shown [27] to exist as a monomer. However, one attempt to terminate the reaction at this stage led only to tarry products.

The formation of the decafluoroazoxybenzene as the main product seems to indicate the relative ease of the oxidation of decafluoroazobenzene (orange stage) with excess hydrogen peroxide. This reaction also occurs in the hydrocarbon series. The fact that no pentaflouronitrobenzene was obtained indicates that oxidation is not the sole reaction. In view of the rapid oxidation occurring when 90 percent peroxide was used [26], it would appear that, at the nitroso stage in our work, coupling with unused pentafluoroaniline predominates over further oxidation of the nitroso derivative. These reactions are illustrated as follows:

**Figure:**
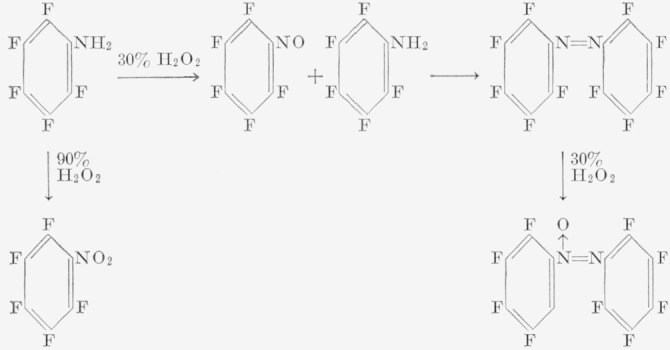

The decafluoroazobenzene can be readily prepared from the azoxy compound by reduction with zinc and ammonium chloride in methanol. Traces of pentafluoroaniline and an additional compound (which may be the decafluorohydroazobenzene) were also obtained.

### 2.4. Reactions With Organolithium Compounds

In some earlier work from this laboratory, it was found that the Grignard reagent, methylmagnesium bromide, reacts with hexafluorobenzene to produce 2,3,4,5,6-pentafluorotoluene. Under the conditions described [18], the yield was low (3%). Alkyl-, alkenyl-, and aryl-lithium reacted readily to give the corresponding substituted pentafluorobenzenes.

**Figure:**
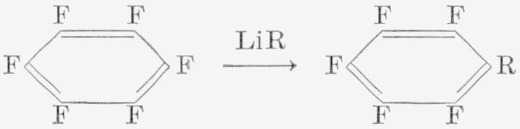

Methyl- and butyl-lithium reacted readily [35], as well as phenyl-, vinyl-, and isopropenyl-lithium [36].

The reactions are rapid, and some di- [35, 36, 37], as well as tri- [26, 35], substituted products were obtained. The preferred method of reaction is usually the addition of a solution of the lithium reagent to a solution of the aromatic fluorocarbon. Even so, disubstitution readily occurs. In the methyllithium reaction, 2,3,5,6-tetrafluoroxylene was identified [37], indicating that the second displacement of fluorine is *para* to the first. The yields of the pentafluorotoluene and 2,3,4,5,6-pentafluorobiphenyl reach 70 percent or higher. Butyllithium has so far given lower yields, but this reagent has been studied relatively little. The alkenyllithium reactions gave yields of the order of 30 to 50 percent, but this is the result of the further reaction of the products to give polymeric solids [38]. The lithium reagents themselves are good catalysts for the anionic polymerization of olefins.

2,3,4,5,6-Pentafluorotoluene can be halogenated to the corresponding benzyl chloride and bromide [37]. With chlorinating reagents, ring addition of halogen interferes with the production of the benzotrichloride [39]. The benzyl halides are versatile reagents for further synthesis of fluoroaryl compounds [37, 39].

In the course of our investigations of the reactions of hexafluorobenzene with organolithium compounds, it was decided to study the reaction of the simplest nucleophile, the hydride ion, with hexafluorobenzene. It was found that hexafluorobenzene does indeed undergo nucleophilic attack by the hydride ion to give pentafluorobenzene as the major product (together with traces of tetrafluorobenzene). The extent of conversion seems to depend on the proportion of lithium aluminum hydride used.

The reaction apparently occurs only when lithium aluminum hydride is the source of the hydride ions. When lithium hydride was employed, no formation of pentafluorobenzene or other products was observed. However, if the lithium hydride was used together with a small quantity of lithium aluminum hydride as a hydrogen carrier [40], a 25-percent conversion to pentafluorobenzene was observed. Again, a very small proportion of disubstitution product was formed; this product was presumably 1,2,4,5-tetrafluorobenzene, since this compound has been reported to be formed [40] when pentafluorobenzene is treated with lithium aluminum hydride. In the latter case, in addition to the tetrafluorobenzene, a high-boiling liquid is obtained in about 5-percent yield; the residue distils in the range of 80 to 20 °C at 5 mm. (Direct replacement of fluorine atoms by hydrogen atoms on the aromatic nucleus can also be accomplished by the use of hydrogen over platinum-on-carbon at 300 °C [17].)

### 2.5. Mechanism of Reaction

In this work, the reactivity of hexafluorobenzene toward a great variety of nucleophilic reagents has been explored. No specific investigations of the mechanistic features of the reactions involved were carried out, and the details of the various mechanisms must be left for future studies. No single mechanism is likely to be adequate for all of the reactions reported. In fact, the variety of conditions and reagents examined render it extremely likely that a variety of mechanisms is involved in the reactions reported.

The mechanisms of the nucleophilic reactions of aromatic systems have been reviewed [42, 43, 44]. The effects of solvents on these reactions has, apparently, not yet been investigated in great detail. However, review [45] of the effects of solvation on the properties of anions in dipolar, aprotic solvents discusses many aspects of nucleophilic mechanisms and the role of the solvent in them. In our work a variety of solvents, protic and aprotic, were utilized.

Although no specific kinetic work was carried out, the large amount and variety of results obtained allow certain mechanistic concepts to be developed. It seems evident, for instance, that a benzyne intermediate is not involved in our reported reactions of hexafluorobenzene in which a monosubstituted pentafluorobenzene is produced (even when it is treated with sodamide in liquid ammonia). This reaction of hexafluorobenzene is relatively efficient and is encumbered chiefly by the reactivity of the aniline product, which can react (via the anilide ion) to afford such products as perfluorodiphenylamine [25]. Tetrafluorobenzyne has, however, been reported [46] as a decomposition product of lithium pentafluorophenyl prepared from lithium amalgam and brornopentafluorobenzene. It is apparently stable but quite reactive. It would seem that benzyne formation in alkali-metal amide reactions in liquid ammonia is a secondary aspect of these nucleophilic reactions, although it explains various isomerizations observed in systems containing aromatic hydrogen atoms.

The reactions of hexafluorobenzene with alkali-metal hydroxides and alkoxides, sodamide, and organolithium compounds presumably involve attack on the hexafluorobenzene by negative ions, and it seems evident that, as a rule, unimolecular ionization of the hexafluorobenzene does not occur. The preparation of pentafluorophenol by treating potassium hydroxide in *text*-butyl alcohol with hexafluorobenzene, and the preparation of benzyloxypentafluorobenzene using sodium benzylate in *tert*-butyl alcohol, indicate this; otherwise, it would be expected that *tert*-butyl pentafluorophenyl ether would have been produced. The use of alcoholic and aqueous amine solutions to produce various anilines in which moieties from the solvents are not part of the final products also indicates absence of any appreciable ionization. Furthermore, no reaction occurs between hexafluorobenzene and pure water up to 300 °C, the highest temperature studied. At the higher temperature, addition of a drop of pyridine led to the rapid formation of tarry material, which gave a phenolic odor on acidification; the quantities of tar or char were approximately proportional to the pyridine added. The effect of aqueous potassium cyanide on hexafluorobenzene was also studied in the temperature range of 150 to 250 °C. Only at 250 °C did a reaction occur. However, no identified products were obtained, but only intractable chars. The reaction was violent and was essentially a contained explosion. Extensive damage to the pressure vessel occurred in one instance, although without violent fragmentation of the vessel. A similar study of the effect of aqueous sodium carbonate showed no reaction up to 250 °C, at which temperature, there were formed extensive tars which again gave a phenolic odor on acidification.

Specific complexes probably play an important role in the reported reactions of hexafluorobenzene. It has been observed that a 1:1 molar mixture of benzene, mp 5.4 °C, and hexafluorobenzene, mp 5.0 °C, forms a solid melting at 23.7 °C [47], which demonstrates the formation of a molecular complex between the two species. Freezing-point data indicate complex-formation between hexafluorobenzene and fluorobenzene also, but not between pentafluorobenzene and benzene [47]. These complexes are presumably similar to the charge-transfer complexes known to form between picric acid and certain aromatic compounds. Benzene and 1,3,5-trinitrobenzene, for instance, form a complex [48] which is certainly of a similar character. With more electronreleasing substituents on a benzene nucleus, a more stable complex should form, as with hexafluorobenzene. In the case of mesitylene and hexafluorobenzene, a complex melting at 24 °C has been observed; one melting at 56 °C is formed between hexafluorobenzene and 2-methylnaphthalene [47]. These observations point to a specific complex-formation as an explanation of the role of pyridine in enhancing the reactivity of hexafluorobenzene in the reactions reported here. In aqueous systems, pyridine may be effecting simple ionization of the hexafluorobenzene.

In the reactions of hexafluorobenzene with ammonia or primary and secondary amines, the mechanism is probably one in which the un-ionized amine directly attacks the fluoroaromatic molecule:

**Figure:**
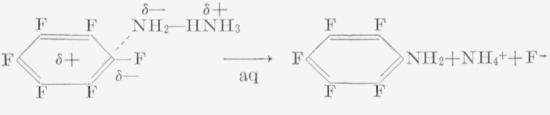

Whether the second ammonia molecule is nearby or not appears to be unimportant; a transitory hydronium ion would be equally effective.

The action of neutral water species, although obviously much less effective than nitrogen compounds, is likely to be involved to some extent in the formation, for instance, of pentafluorophenol by the reaction of aqueous alkali hydroxides with hexafluorobenzene.

By analogy with the formation of such complexes as that [42] formed by the addition of a methoxide ion to 2,4,6-trinitrophenetole, it is likely that hexafluorobenzene in its reactions with the amide, hydroxy, alkoxy, and alkyl anions forms a relatively stable, transition state.

A somewhat startling and puzzling facet of the reactions of fluorobenzene derivatives is the splitting of the alkoxypentafluorobenzenes by aqueous ammonia or hydrazine [20] and, presumably, by alcoholic potassium hydroxide [18]. In the hydrazine reaction, the hydrazine salt of pentafluorophenol is obtained. This reaction has been postulated to occur via a splitting of the methyl—oxygen link [20], presumably, as shown in the following series of reactions.

**Figure:**
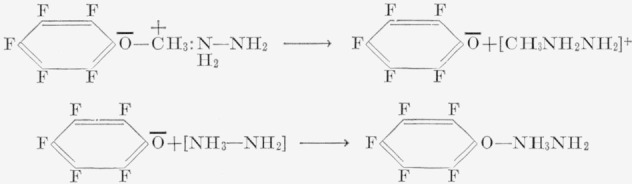

This splitting has only been observed in the presence of water, and is not necessarily related to the basicity of the medium; it does not occur in the liquid ammonia—sodamide reaction, where a fluorine atom is replaced without splitting of the ether link. However, sodamide in liquid ammonia does split anisole. Thus, it is possible that the split occurs at the pentafluorophenyl—oxygen link, and that water is the key reagent. For the reaction to occur at the phenyl—oxygen link, one can logically postulate either an attack by the hydroxide ion, which implies that the ring carbon atom at this site is highly positive, or some direct reaction at this carbon atom with a neutral water molecule. The former implies easier ionization of the phenyl— oxygen link, and not necessarily an enhancement of the positive character of the ring carbon atom linked to the oxygen atom. It is conceivable that the methyl—oxygen link is quite covalent. Since water was present in all of the reactions in which cleavage of the ether moiety was observed, it seems feasible that simple hydrolysis may have occurred. However, distilled water failed to react with pentafluoroanisole under conditions identical with those under which aqueous amine bases produced cleavage. Thus, the role played by the water molecules may simply be as a co-catalyst with the base, to aid polarization of the ether linkage, in much the manner suggested previously for pyridine. Determination of the bond actually ruptured would require experiments with H_2_O^18^.

## 3. Identification of Isomers

The orientation of the isomers of most of the difunctional derivatives of hexafluorobenzene of the general formula, C_6_F_4_*XY*, where *X* and *Y* may be similar or dissimilar groups (but not a fluorine atom), has been determined by nuclear magnetic- resonance spectroscopy. Direct syntheses of these derivatives for comparison and identification purposes by alternative routes has been difficult, although this has been accomplished in several cases [22, 25]. Infrared and ultraviolet spectroscopy have so far not found extensive use in identifying structural isomers of aromatic fluorocarbons. In a series of articles, Baker [49, 50] has described the vibrational spectra of *o*-halophenols in the nearinfrared region; these compounds show a doubling of the hydroxyl stretching frequencies at low concentrations. This doubling of the hydroxyl band is absent for *para*-substituted halophenols and presumably for *meta*-substituted halophenols, although slight shifts of hydroxyl frequency do occur. Therefore, this method appeared to be a useful supplement to nuclear magnetic-resonance in distinguishing between the isomeric phenols obtained from the reaction of various pentafluorohalobenzenes with alkali hydroxides. The procedure appears to be specific for *o*-halophenols, in particular when the halogen atoms differ, because of the two types of hydrogen bonding that can occur. For example, the structures applied in our case to the 2-bromo-3,4,5,6-tetrafluorophenol are:

**Figure:**
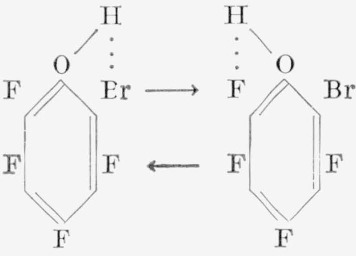

**Figure 1:**
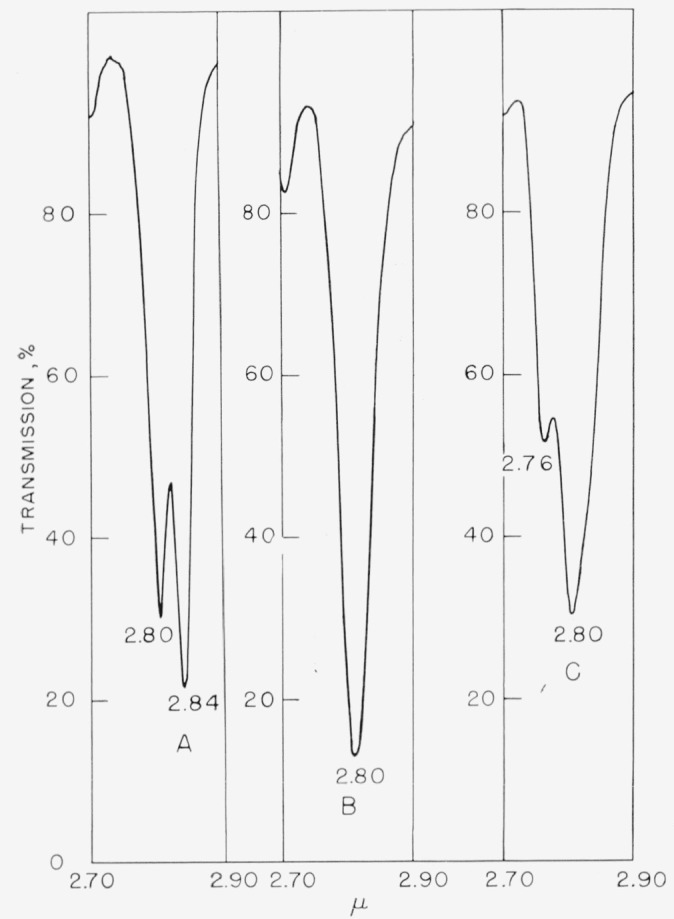
The *OH* spectra of halophenols in carbon tetrachloride. 2-Bromo-3,4,5,6-tetrafluorophenol;2-Chloro-trifluoro-*α*,*α*,*α*-trifluoro-*p*-cresol;2-Chloro-trifluoro-*α*, *α*,*α*-trifluoro-*o*-cresol.

In figure 1-A, this compound exhibits the split hydroxyl band indicative of different halogen atoms, surrounding the hydroxyl group at the *ortho* positions. In this case, the Δ*v* OH for fluorine is at 2.80 *µ*, and the Δ*v* OH for bromine at 2.84 *µ* is in agreement with values shown by Baker [50]. This doublet is absent from, and only a lone hydroxy band appears in, the spectrum of 4-bromo-2,3,5,6-tetrafluorophenol at 2.80 *µ*. However, since the 3-bromo-2,4,5,6-tetrafluorophenol is unknown at present, it is imposssible to ascertain whether or not the (large) bromine atom at this position would cause some steric effect on the *ortho* fluorine atom so as to change the electron environment whereby a doublet may be obtained instead of the single band. However, as expected, various tetrafluoro- phenols having substituents in the *para* position show the single hydroxyl band at 2.80 to 2.81 *µ*, similar to those shown in figure 1-B. Some of these phenols are: 2,3,5,6-tetrafluorophenol, 2,3,4,5,6- pentafluorophenol, 2,3,5,6-tetrafluoro-p-cresol, and 2,3,5,6-tetrafluoro-4-iodophenol. By reverse reasoning, therefore, the absence of the split hydroxyl band in the near-infrared region in these simple halophenols is indicative that groups other than fluorine atoms are not *ortho* to each other. These compounds were further confirmed by nuclear magnetic-resonance spectroscopy to be *para* isomers.

An attempt to extend this method of distinguishing isomeric phenols to a more complex molecule has met with partial success. For example, in figures 1-B and 1-C are shown the spectra of two phenolic products obtained from the reaction of 2-chloro-3,4,5, 6-tetrafluoro-*α*,*α*,*α*-trifluorotoluene with potassium hydroxide in pyridine. Since, in figure 1-B, only the single hydroxyl band appears, it is obvious that the hydroxyl group is located in either the 4 or the 5 position. From other considerations, the trifluoromethyl group would be expected to exert a greater directive effect than the chlorine atom. In this case, then, the hydroxyl group is probably located *para* to the trifluoromethyl group. However, in figure 1-C, the hydroxyl group would appear to be *ortho* to the trifluoromethyl group, since the Δ*v* OH for chlorine would be expected to be at a higher wavelength (at 2.82 *µ*). The band at 2.76 *µ* may in this instance again arise from the change in frequency of the hydroxyl group because of the two types of hydrogen bonding possible, for example, a five-membered bridge (I) versus a six-membered bridge (II), as shown below:

**Figure:**
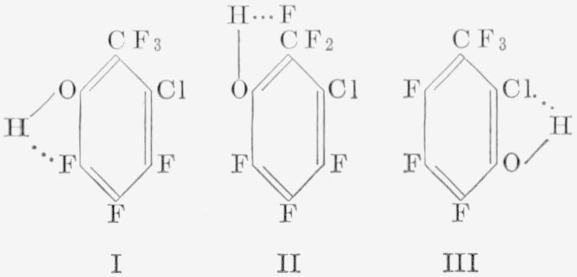

It could be argued that this band may be attributable to a free (*trans*) hydroxyl group, such as occurs with other *ortho*-monohalogenated (Cl, Br, I) phenols, but this doubling has not been observed in *o*-fluorophenol [49]. Similarly, the vapor infrared spectrum of pentafluorophenol has a strong band at 2.75 *µ* attributed to a free hydroxyl group [19], but, in a nonpolar solvent such as carbon tetrachloride, the band is shifted to a higher wavelength (at 2.80 *µ*). Therefore, the band observed in figure 1-C at 2.76 *µ* is probably associated with the *o*-trifluoromethyl group, and not with a free hydroxyl group or an *o*-chlorine atom. On this basis, then, tentative identification of 3-chloro-4,5,6-trifluoro-2-(*α*,*α*,*α*-trifluoromethyl) phenol has been made, and the compound has been assigned the structures shown in I and II.

**Figure 2:**
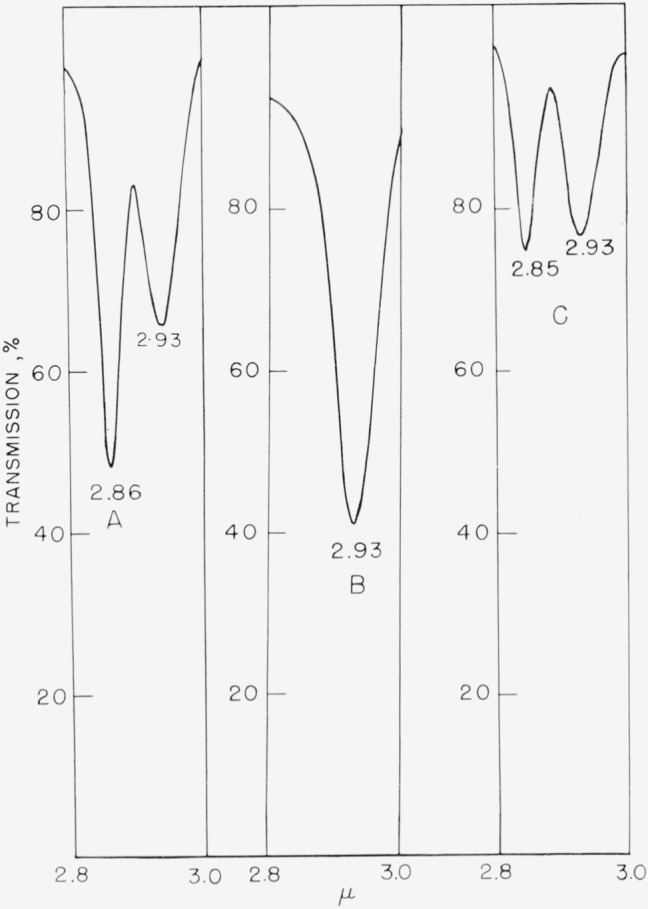
The *NH_2_* and *NH* spectra of haloanilines in carbon tetrachloride. 2,3,4,5,6-Pentafluoroaniline;2,3,5,6-Tetrafluoro-1,4-bis(*N*-methylamino) benzene;2-Chloro-trifluoro-*α,α,α*-trifiuoro-*p*-toluidine.

Attempts to apply this “*ortho* effect” to other derivatives of hexafluorobenzene, particularly to the aniline compounds, has been fruitless. For example, in the near-infrared region, 2,3,4,5,6-pentafluoroaniline shows two distinct peaks, at 2.86 *µ* and 2.93 *µ* (fig. 2-A). This is also true of 3-chloro-2,5,6-trifluoro-4-(*α*,*α,α*-trifluoromethyl)aniline, figure 2-C. In these aniline derivatives, the bands appear to be associated with the number of hydrogen atoms on the nitrogen atom. Replacing one of the hydrogen atoms by a methyl group merely eliminates one of the bands. This is also evident in figure 2-B, even though the compound contains two methylamino groups. Further evidence is obtained from the fact that 2,3,4,5,6-pentafluoro-*N*,*N*-dimethylaniline does not have an absorption band in this region. However, this near-infrared region does offer the possibility of distinguishing between primary, secondary, and tertiary fluoroanilines.

## 4. Directional Effects

A considerable number of disubstituted (nonfluorine groups) derivatives of tetrafluorobenzene have been prepared and their structures confirmed by unequivocal means. The directional effects determining the site of the second substitution in the electrophilic reaction of benzene has been the subject of much investigation. These effects are directly related to the charge distribution in the monosubstituted benzene and can be estimated from dipolemoment data [51]. Dipole moments of some fluorobenzenes have been reported [52]. The compounds studied do not, however, include any pentafluorobenzenes. The present work affords an insight into the character of the directional effects in polyfluorobenzenes. In table 1 are summarized these effects as observed from the reactions of various bases with a variety of monosubstituted pentafluorobenzenes. For example, the reaction of pentafluorobenzene, where R = H, with potassium hydroxide yields the 2,3,5,6-tetrafluorophenol. On further examination of table 1, it is apparent that, from the three possible isomers, the *para* product was obtained in most cases. This was true even though a great variety of conditions was used. It seems, therefore, that the isomer finally obtained depends on a number of involved parameters rather than on any single condition. From table 1, it is also evident that, in those cases where very reactive nucleophiles (such as the amide ion, lithium reagents, or alkoxides) were used, the *para* product was always isolated. These bases react to form salts with such functional groups as amines or hydroxyl groups, and no displacement of fluorine occurs.

With less reactive nucleophiles, such as aqueous hydroxides, ammonia, or alkylamines, isomers other than the *para* were *also* isolated. However, in these reactions, the isomer obtained probably depends to a large degree on the group (R) *already* present in the molecule, as well as on the nucleophile used. For example, the nitro group in pentafluoronitrobenzene exerts a powerful influence on the *ortho* positions, and, on reaction with ammonia, gives almost theoretical yields of *o*- and *p*-nitroamines in 7:3 ratio. Conversely, the amino group in pentafluoroaniline exhibits a strong influence in nucleophilic reactions at the *meta* position; as a result, the *m*-diamine is, with ammonia, the primary reaction-product. But the reaction of methylamine with pentafluoro-*N*-methylaniline yields only the *para* isomer, whereas the reaction of pentafluoro-*N*,*N*-dimethylaniline with dimethylamine yields the *para*, *meta*, and *ortho* isomers in a 10:4:1 ratio. Although the reactions of these amines appear to be “anomalous,” a point could be made of the fact that the methyl- and dimethylamines are stronger nucleophiles than ammonia. In this case, then, the *para* structure would be the expected predominant isomer, which, indeed, it was. Similarly, it has been observed that the reaction of pentafluoro-*N,N*-dimethylaniline with ethoxide ion results in only the *para* product. It is likely that not only the attacking nucleophile but also the resonance effects (due to nonfluorine groups) determine the isomer(s) that are obtained. The ease of fluorine displacement is probably increased by the ability of the solvent molecudes to localize the electron density around this fluorine atom. Consideration must also be given to the possibility that resonance effects in the ground state may be altered in the transition state by the relative reactivity of the attacking nucleophile, thereby making possible the attack at a position other than the preferred site. For example, consider the following two structures from simple charge [2] effects:

**Figure:**
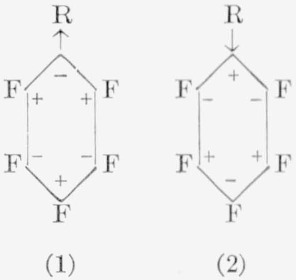

In (1), the incoming anion, — R_1_, would be expected to attack at the electron-deficient *ortho* and *para* positions, giving rise to either *ortho* or *para* products. In (1), if R=NO_2_, the ground state could be visualized as shown, and the transition state as either (3) or (4).

**Figure:**
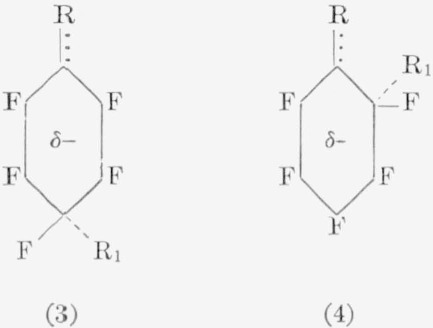

The *o*- and *p*-nitroamines obtained indicate occurrence of this mode of reaction for pentafluoronitrobenzene, and it is doubtful whether use of a stronger nucleophile would alter the outcome. In (2), if R = NH_2_ (an electron-donating group), the *meta* position relative to R appears to be the preferred site, and *meta* products would be expected. With a base such as ammonia, this is indeed the case. From consideration of the possible resonance structures (5) to (7a), it would appear that (7) is the predominant reacting species with a weak base, since the *meta* positions

**Figure:**
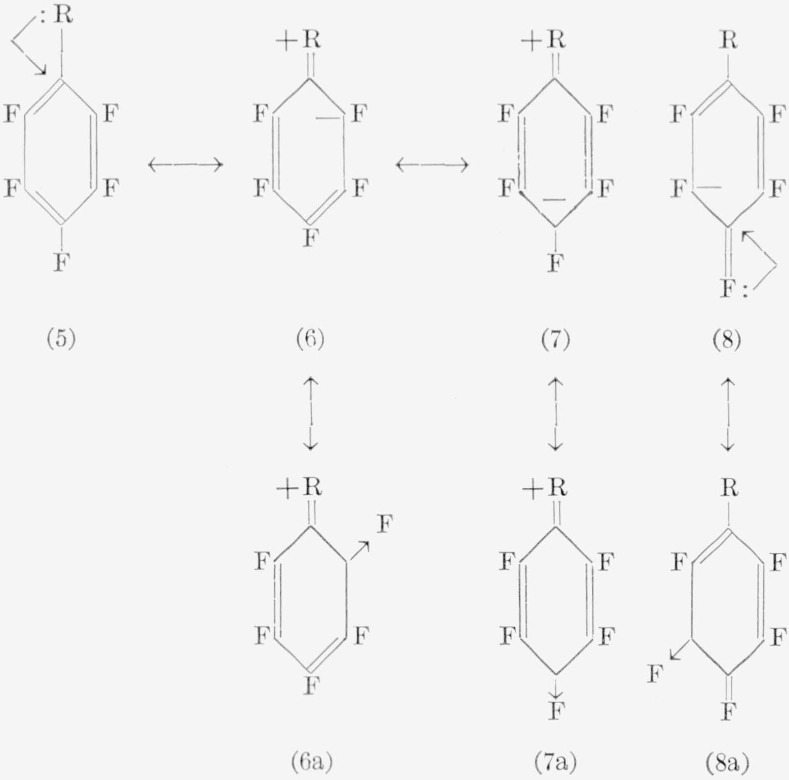

appear least negative. An alternative explanation could be offered in that a resonance structure, as in (8a), could be the reacting species. The induced charge would “prefer” to reside closer to the fluorine atom than to the carbon atom, and this effect would favor separation of a fluoride ion. Reaction would then be expected to occur at this site, giving rise to *meta* products. Effects similar to these have been used to explain the decrease in acidity of *p*-fluorophenol [53]. On the other hand, following similar reasoning, the resonance form (7a) would be expected to afford *para* products. This structure (7a) again appears to be the reacting species in the reaction of pentafluoro-*N*-methylaniline with methylamine, because only the *para* isomer was isolated. All three isomers were obtained with dimethylamine and pentafluoro-*N*,*N*-dimethylaniline. This indicates much less selectivity in structures (6a), (7a), and (8a), although the para isomer was obtained in greater proportions (somewhat surprisingly, since methylamine and dimethylamine have about equal basicity). In several other reactions also, all three isomers have been detected. Refluxing of pentafluorobenzene with lithium aluminum hydride in ether produces *o*:*m*:*p* isomers of tetrafluorobenzene in 7:1:92 ratio, the orientational prefixes referring to the relative positions of the *hydrogen* atoms [54]. On treating pentafluorobenzene with ethanolic hydrazine, an *o*:*m*:*p* distribution of 6:1:93 is obtained [52]. Sodium hydrogen sulfide reacts with pentafluorobenzene in a glycol—pyridine mixture to yield only 2,3,5,6-tetrafluorothiophenol [55].

The reactions of pentafluoroanisole with bases yield additional information regarding directional effects of the anilines, since the amino and methoxyl groups have nearly equivalent influence on electrophilic substitution. However, the reaction of pentafluoroanisole with aqueous ammonia causes cleavage of the ether moiety. Resonance structures, such as (9), probably aid in the cleavage reaction.

**Figure:**
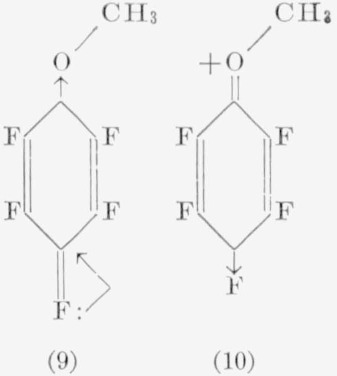

Other factors (such as solvent, and the ability of various groups to coordinate with the oxygen atom) also play an important role. The reaction with aqueous bases, therefore, simply becomes a competition between cleavage and replacement. With strong nucleophiles, such as amide ion or methoxide ion, only substitution occurs, presumably through a resonance form (10). In fact, such structures as (6a), (7a), and (10) appear to explain the majority of the products observed in nucleophilic reactions of aromatic fluorocarbons. For, if such forms as (8a), (6), and (7) were major contributing resonance forms, it should be possible to isolate *meta* products from most of the reactions encountered, which has not been the case. The effects, in general, do not show any specific differences qualitatively incompatible with the usual interpretations of directional effects in electrophilic reactions of monosubstituted benzenes. The predominance of *para* substitution suggests that the most important effect is simply an inductive one, since few of the monosubstituents can compete in electron withdrawability with fluorine. A much greater knowledge of the mechanisms of these reactions is needed before the directional effects can be satisfactorily classified.

## 5. Experimental Procedure

### 5.1. Reaction With Hydroxides

#### a. 2,3,4,5,6-Pentafluorophenol

In a 188-ml bomb were placed 40 g (0.207 mole) of hexafluorobenzene, 26.5 g (0.39 mole) of 85 percent potassium hydroxide, and 75 ml of distilled water. The bomb was sealed and heated at 175 °C for 5 hr with agitation. It was then cooled and opened, and the contents were filtered. The white salt of potassium pentafluorophenoxide hydrate was acidified with dilute hydrochloric acid, and the pentafluorophenol was separated from the aqueous phase. The aqueous solution was extracted several times with 50-ml portions of dichloromethane. The organic layers were combined, dried (sodium sulfate), evaporated, and distilled. After removal of the solvent, there was obtained 33.1 g (83.5%) of pentafluorophenol, bp 144 to 145 °C.

#### b. 2,3,5,6-Tetrafluorophenol

In a 500-ml, three-necked flask were placed 67 g (0.4 mole) of pentafluorobenzene, 21.6 g (0.4 mole) of potassium hydroxide, 150 ml of pyridine, and 2 ml of water. The contents were heated to reflux with vigorous stirring, and reflux was continued for 1 hr after the hydroxide had dissolved. Then, 21.6 g (0.4 mole) of potassium hydroxide was added through the condenser, and heating and stirring were continued for 24 hr. The pyridine was removed by distillation under reduced pressure, and the residue was recrystallized twice from water, yielding 70 g of potassium tetrafluorophenoxide dihydrate. The salt was acidified with dilute hydrochloric acid, the phenol was separated and dissolved in dichloromethane, and the solution was dried (sodium sulfate), evaporated, and distilled under reduced pressure, bp 47 °C/20 mm. Yield: 21 g (32% based on pentafluorobenzene). The structure of the compound was verified by nuclear magnetic-resonance spectra due to fluorine atoms.

*Analysis:* Calculated for C_6_H_2_F_4_O: C, 43.3; H, 1.2. Found: C, 43.5; H, 1.5.

#### c. 2,3,5,6-Tetrafluoro-*p*-cresol

In a 500-ml, three-necked flask, a mixture of 36 g (0.2 mole) of pentafluorotoluene, 28 g (0.5 mole) of potassium hydroxide, and 300 ml of *tert*-butyl alcohol was refluxed, with stirring, for 2 hr. The flask was cooled and 100 ml of water was added, after which 100 ml of the alcohol was distilled off. This process was repeated until all of the alcohol had been removed, the temperature of the takeoff rising from 73 to 100 °C. The distillation was continued until 6 g of pentafluorotoluene had been recovered. The residue was made acid with dilute hydrochloric acid, cooled, extracted with three 100-ml portions of ether, and the extracts were dried (sodium sulfate) overnight. After evaporation of the ether, the black, oily residue was sublimed twice, yielding 12 g (32%) of white crystals; mp 52 °C. Fluorine and proton absorption in the nuclear magnetic-resonance spectra confirmed the structure.

*Analysis:* Calculated for C_7_F_4_H_4_O: C, 46.6; H, 2.2. Found: C, 46.3; H, 2.4.

#### d. 2,3,5,6-Tetrafluoro-4-iodophenol

To 20 g (0.068 mole) of pentafluoroiodobenzene in 100 ml of pyridine were added 2 g (0.136 mole) of potassium hydroxide and 1 ml of water, and the mixture was refluxed for 4 hr. At the end of this period, an additional 2 g of potassium hydroxide was added and the mixture was refluxed for an additional 8 hr. After being cooled, 300 ml of 10% aqueous hydrochloric acid was added, and the crude, yellow tetrafluoroiodophenol (10.1 g) was removed by filtration. Extraction of the aqueous layers with two 100-ml portions of dichloromethane gave an additional 2 g of the phenol. Net yield (based on pentafluoroiodobenzene, 1.5 g of which was recovered): 66 percent.

The tetrafluoroiodophenol was recrystallized from ether—petroleum ether as white plates, mp 79 to 81 °C. Nuclear magnetic resonance showed this to be the *para* isomer; no other isomer was isolated. The near-infrared absorption spectrum showed only one hydroxyl peak, at 2.81 *µ*.

The benzoate was prepared in the usual way. Recrystallization from ether—petroleum ether, followed by vacuum sublimation (60 °C/1 mm), gave white microcrystals, mp 59 to 60.2 °C.

*Analysis:* Calculated for C_13_H_5_F_4_IO_2_: C, 39.41; H, 1.26; F, 19.1; I, 32.04. Found: C, 40.0; H, 1.35; F, 18.8; I, 31.9.

#### e. Bromotetrafluorophenols

The preparation of the bromotetrafluorophenols is essentially the same as that for the tetrafluoroiodophenol, except that the following quantities were used: 66 g (0.26 mole) of bromopentafluorobenzene, 150 ml of pyridine, 1 ml of water, and 28.5 g (0.53 mole) of potassium hydroxide. After acidification of the reaction mixture, the products were extracted with three 100-ml portions of dichloromethane. The combined extracts were dried (sodium sulfate) and the solvent was evaporated. Vacuum distillation of the residual liquid gave 10.5 g (16%) of unreacted bromopentafluorobenzene and two phenolic fractions.

*Fraction 1:* 31 g (56.3%); bp 73 to 75 °C/2 mm; mp 19 to 21 °C; was shown by analytical, vaporphase chromatography (Viton A column at 175 °C) to be a mixture of two components in a 3:1 ratio.

One component was 2-bromo-3,4,5,6-tetrafluorophenol, 3.5 g (6.3%), mp 41 to 43 °C. Nuclear magnetic-resonance spectra showed this compound to be the *ortho* isomer. This is also evident from the near-infrared spectra, which showed a split hydroxyl peak at 2.81 *µ* and 2.84 *µ* presumably due to the two types of hydrogen bonding that can occur.

The 3,5-dinitrobenzoate had mp 104 to 105 °C.

*Analysis:* Calculated for C_13_H_3_BrF_4_N_2_O_6_: C, 35.6; IT, 0.69; Br, 18.1. Found: C, 35.7; H, 0.7; Br, 18.7.

The second (and main) component of fraction 1 was 4-bromo-2,3,5,6-tetrafluorophenol, 11.5 g (20.9%). The 3,5-dinitrobenzoate had mp 131 to 133 °C.

*Analysis:* Calculated for C_13_H_3_BrF_4_N_2_O_6_: C, 35.6; H, 0.69; Br, 18.1. Found: C, 35.4; H, 0.6; Br, 20.3.

*Fraction 2:* 4 g (7.2%); bp 90 to 92 °C/2 mm; mp 58 to 60 °C; was shown by nuclear magnetic resonance to be 4-bromo-2,3,5,6-tetrafluorophenol. Also, the near-infrared absorption spectrum showed only one peak in the 2.8-*µ* region. The 3,5-dinitrobenzoate had mp 133 to 134 °C. There was no depression of melting point on mixing the 3,5- dinitrobenzoate of this fraction with that of the second component of fraction 1.

#### f. 2-Chlorotrifluoro-*α*, *α, α*-trifluoro-*o*-(and-*p*-)Cresols

To 100 g (0.4 mole) of 2-chlorotetrafluoro-*α*,*α*,*α*- trifluorotoluene in 100 ml of pyridine were added 45.6 g (0.8 mole) of potassium hydroxide and 1 ml of water. The mixture was slowly heated to 90 °C for 2 hr with stirring, cooled, poured into 1 liter of 20 percent sulfuric acid, and allowed to settle overnight. It was filtered, and the bottom (fluorocarbon) layer was separated from the aqueous filtrate. The aqueous layer was further extracted with several 100-ml portions of ether. The combined organic layers were dried (sodium sulfate) and the solvent removed, yielding 38 g (38.6%) of a mixture of two isomeric cresols, bp 97 to 103 °C/15 mm. The components were separated by preparative vapor-phase chromatography using a Viton A column at 190 °C. The first compound eluted was 2-chlorotrifluoro-*α*,*α,α*-trifluoro-*o*-cresol: 0.5 g; bp 92 to 93 °C/15 mm; $n_{D}^{24}$ 1.4510; infrared absorption showed a split hydroxyl band, at 2.76 *µ* and 2.81 *µ* The second compound was 2-chlorotrifluoro-*α*,*α*-trifluoro-*p*-cresol: 15 g; bp 102 to 103 °C/15 mm; $n_{D}^{24}$ 1.4510; infrared absorption showed only one (strong) hydroxyl band, at 2.81 *µ*.

### 5.2. Reactions With Alkoxides

#### a. 4-Bromo-2,3,5,6-tetrafluoroanisole

A solution of 11.5 g (0.5 g-atom) of sodium in 150 ml of anhydrous methanol was added dropwise to a stirred solution of 123.5 g (0.5 mole) of bromopentafluorobenzene in 70 ml of dry pyridine at reflux temperature. The addition required 1.5 hr; refluxing was continued for an additional 15 hr. The mixture was cooled and poured into 1 liter of 10 percent aqueous hydrochloric acid. The usual isolation, as described above, gave 89 g (68.8%) of bromotetrafluoroanisole, bp 76 to 79 °C/5 mm. Analytical vapor-phase chromatography (Viton A column at 175 °C) showed slight contamination of the product with another compound. Separation was accomplished by preparative scale vapor-phase chromatography, using 10-g samples (Viton A column at 175 °C). The fore-cut, 8 g, still gave two peaks. The (main) second fraction, 66 g, showed only one peak in analytical vapor-phase chromatography; bp 79 to 81 °C/5 mm; $n_{D}^{25}$ 1.4812. Nuclear magnetic resonance showed this to be the *para* isomer.

*Analysis:* Calculated for C_7_H_3_BrF_4_O: C, 32.4; H, 1.2; Br, 30.9. Found: C, 32.7; H, 1.3; Br, 31.9.

A higher-boiling fraction (2 g), 72 to 76 °C/2 mm, had three components; these were not investigated further.

#### b. 2,3,5,6-Tetrafluoro-4-iodoanisole

To a stirred solution of 10 g (0.034 mole) of pentafluoroiodobenzene and 50 ml of dry pyridine, heated to reflux temperature, was added a solution of 0.8 g (0.034 g-atom) of sodium in 15 ml of anhydrous methanol. After being refluxed for 3 hr, the cooled solution was poured into 100 ml of 6 *N* aqueous hydrochloric acid. The orange (fluorocarbon) layer was separated, and the aqueous layer extracted twice with 50-ml portions of ether. The combined organic layers were dried (sodium sulfate), evaporated, and distilled. After removal of the unreacted pentafluoroiodobenzene (1.5 g), there was obtained 5.5 g (63% based on reacted pentafluoroiodobenzene) of 2,3,5,6-tetrafluoro-4-iodoanisole; bp 113 to 115 °C/20 mm; $n_{D}^{22}$ 1.5229; the structure was confirmed by nuclear magnetic resonance. Analytical, vapor-phase chromatography (at 190 °C) showed a 3-percent contamination with starting material. The sample was redistilled before elementary analysis.

*Analysis:* Calculated for C_7_H_3_F_4_IO: C, 27.48; H, 0.98; F, 24.83; I, 41.48. Found: C, 27.8; H, 1.0; F, 24.4; I, 41.0.

#### c. Octafluoro-4,4′-dimethoxybiphenyl

A mixture of 1 g of 2,3,5,6-tetrafluoro-4-iodoanisole and 1 g of activated copper powder was gently refluxed for 12 min, cooled, and extracted first with 10 ml of acetone and then with 10 ml of benzene. The combined extracts were filtered and the solvents evaporated. The yellow solid obtained was decolorized with charcoal, and recrystallized from petroleum ether. There was obtained 0.2 g (17%) of octafluoro-4,4′-dimethoxybiphenyl as white plates; mp 90 to 91.2 °C.

*Analysis:* Calculated for C_14_H_6_F_8_O_2_: C, 46.94; H, 1.69; F, 42.44. Found: C, 47.5; H, 1.88; F, 42.8.

#### d. Benzyl Pentafluorophenyl Ether

##### Method A

In a 500-ml flask equipped with stirrer and reflux condenser, 9 g (0.4 g-atom) of sodium was treated with 250 ml of benzyl alcohol, and the solution cooled overnight. Then, 75 g (0.41 mole) of hexafluorobenzene was added, stirring was resumed, and the mixture slowly heated to the reflux temperature of hexafluorobenzene. Heating and stirring were continued for 24 hr, and then the mixture was cooled overnight with stirring. The precipitate was filtered off with suction, the filtrate was concentrated, ethanol was added, and the resulting solution was refrigerated. The resulting precipitate was recrystallized until a constant melting point (44 °C) was obtained; yield, 30 g (28%) of white crystals.

##### Method B

In a 500-ml flask, 4.6 g (0.2 mole) of sodium was added to a solution of 22 g of benzyl alcohol in 250 ml of *tert*-butyl alcohol. The solution was cooled overnight and 40 g of hexafluorobenzene (0.22 mole) was added. The solution was stirred and refluxed for 40 hr, after which the alcohols were distilled off. The product was recrystallized twice from ethanol; yield, 33 g (60%): mp 44 °C.

*Analysis:* Calculated for C_13_F_5_H_7_O: C, 56.9; H, 2.6. Found: C, 56.8; H, 2.6.

#### e. 2,3,4,5,6-Pentafluorophenyl Ether and 2,3,5,6-Tetrafluoro-1,4-diphenoxy benzene

##### Method A

To 8 g (0.043 mole) of hexafluorobenzene in 30 ml of *N*,*N*-dimethylformamide was added 5.28 g (0.04 mole) of potassium phenoxide in one portion at room temperature. After the initial reaction had ceased, the mixture was heated at 120 °C for 0.5 hr, cooled, and diluted with 100 ml of water. The bottom (fluorocarbon) layer eventually solidified, and the white solid was separated by filtration. After drying, and fractional sublimation at 70 °C/1 mm, there was obtained 3.5 g (31.3%) of 2,3,4,5,6-pentafluorophenyl phenyl ether, mp 28 to 29 °C. This compound was identical with that described under Method B.

There was also obtained 1 g (7%) of 2,3,5,6-tetrafluoro-1,4-diphenoxybenzene as white crystals, mp 147 to 149 °C.

*Analysis:* Calculated for C_18_H_10_F_4_O_2_: C, 64.6; H, 2.99; F, 22.7. Found: C, 64.75; H, 2.98; F, 23.1.

##### Method B

In a 43-ml bomb were placed 11 g (0.05 mole) of potassium pentafluorophenoxide, 15 g (0.10 mole) of bromobenzene, and 1 g of copper which had been activated by Vogel’s method [56]. The bomb was heated to 210 °C with rocking, maintained at this temperature for 6 hr, and rocked overnight as it cooled to room temperature. The mixture was filtered and the filtrate distilled through a short column. After the excess bromobenzene had been removed, 2 g (15.5%, based on the potassium salt of 2,3,4,5,6-pentafluorophenol) of 2,3,4,5,6- pentafluorophenyl phenyl ether was obtained, which boiled at 240 °C and solidified slightly below room temperature.

*Analysis:* Calculated for C_12_H_5_F_5_O: C, 55.4; H, 1.9. Found: C, 55.7; H, 2.1.

#### f. Perfluorophenyl Ethers

To 30 ml of *N*,*N*-dimethylformamide was added 6 g (0.027 mole) of potassium pentafluorophenoxide and 12.8 g (0.069 mole) of hexafluorobenzene; heat was not evolved. After being refluxed for 14 hr, the black solution was poured into 100 ml of water, and the mixture was extracted with several 50-ml portions of ether. The organic layer was separated, dried (sodium sulfate), and evaporated. After the removal of 4.5 g of unreacted hexafluorobenzene, the residual solid was sublimed at 50 °C/50 mm to give 1.5 g (16%) of perfluorophenyl ether; white crystals, mp 67 to 69 °C. Confirmation of the structure was obtained from mass-spectrometer analysis, which showed the parent peak at 350 mass units.

*Analysis:* Calculated for C_12_F_10_O: C, 41.15; F, 54.3. Found: C, 41.53; F, 53.0; H, 0.0.

A second product was obtained from the sublimation; mp 145 to 148 °C. Although its structure has not been confirmed, it may be *p*-bis(pentafluorophenoxy)2,3,5,6-tetrafluorobenzene.

#### g. 4-Ethoxy-2,3,5,6-tetrafluoro-*N*,*N*-dimethylamine

In a 100-ml flask, 0.6 g of sodium was dissolved in 50 ml of absolute ethanol and the solution was refluxed for 1 hr. To this solution was added 5.3 g (0.025 mole) of 2,3,4,5,6-pentafluoro-*N*,*N*-dimethyl-aniline, and the mixture was refluxed for 2 hr and poured into an excess of water. The organic layer was separated, washed with water, dried, and distilled. Yield: 5.1 g (85%) of a clear, colorless liquid boiling at 34 °C/1 mm.

*Analysis:* Calculated for C_10_H_11_F_4_NO: C, 50.6; H, 4.7; N, 5.9; F, 32.1. Found: C, 50.8; H, 5.0; N, 5.9.

### 5.3. Reactions With Amines

#### a. 2,3,4,5,6-Pentafluoroaniline

The reactions using ammonia or amines were carried out in a stainless-steel, silver-lined bomb. A mixture of 280 g (1.5 mole) of hexafluorobenzene and 400 ml of 28-percent aqueous ammonia was sealed in an 800-ml bomb, which was placed in a heater-rocker mechanism previously heated to 235 °C. The bomb was rocked for 2 hr at 235 °C, removed, and rapidly cooled to room temperature in running water. The contents were poured into a large beaker, and the aqueous layer was separated and extracted three times with ether. The ether extracts were dried (sodium sulfate), and evaporated. The substituted aniline obtained in this way was added to the oily layer from the bomb, and the mixture was evacuated in a vacuum desiccator for 8 hr, the volatile materials, mostly hexafluorobenzene and ammonia, being collected in a dry ice—acetone trap. The residual mass of brown crystals was partially sublimed at atmospheric pressure onto a cold-finger condenser, to yield 236 g (86%) of white crystals of pentafluoroaniline; mp 34 °C.

The remainder of residue was heated to about 75 °C at less than 1 mm pressure. White crystals lacking a definite melting point sublimed onto the cold finger. Yield: 28 g (10%). Elementary analysis indicated it to be tetrafluorophenylenediamine, and nuclear magnetic resonance indicated it to be the *meta* isomer contaminated with a small proportion of the *para* isomer.

*Analysis:* Calculated for C_6_H_4_F_4_N_2_: C, 40.0; H, 2.2; N, 15.5. Found: C, 40.2; H, 2.4; N, 15.2.

#### b. 2,3,4,5,6-Pentafluoro-*N*-methylaniline

In a 200-ml bomb, 56 g of hexafluorobenzene and 110 ml of 30-percent aqueous methylamine were heated at 220 °C for 3 hr with continuous rocking. The products were dissolved in ether and the ether was evaported. The pentafluoro-*N*-methylaniline was distilled at atmospheric pressure, and redistilled under reduced pressure. Yield; 59 percent; bp 170 to 172 °C at 760 mm.

*Analysis:* Calculated for C_7_H_4_F_5_N: C, 42.6; H, 2.1; N, 7.1. Found: C, 42.7; H, 2.2; N, 7.0.

The residue was crystallized from absolute alcohol, yielding 2,3,5,6-tetrafluoro-*N*,*N*′-dimethylphenyl- enediamine, which was purified by sublimation and recrystallization. Yield: 25 percent; mp 94 °C.

*Analysis:* Calculated for C_8_H_8_F_4_N_2_: C, 46.2; H, 3.8; N, 13.5. Found: C, 46.1; H, 3.9; N, 13.6.

#### c. 2,3,4,5,6-Pentafluoro-*N*,*N*-dimethylaniline

A mixture of 110 ml of 25-percent aqueous dimethylamine and 50 g of hexafluorobenzene was heated at 235 °C for 2 hr. The reaction products were purified as described for pentafluoroaniline. A colorless liquid which distilled at 88 °C/1 mm was pentafluoro-*N*,*N*-dimethylaniline (65% of the oily layer). Four additional fractions were collected.

*Analysis:* Calculated for C_8_H_6_F_5_N: C, 45.6; H, 2.6; N, 6.6. Found: C, 45.7; H, 2.8; N, 6.5.

*Fraction 2*, bp 88 to 126 °C/1 mm, was identified by vapor-phase chromatography to be a mixture of dimethylaniline with three other compounds. *Fraction 3*, bp 126 to 134 °C/1 mm, contained the three isomers of bis(dimethylamino)tetrafluorobenzene, with the *meta* isomer predominant. *Fraction* 4, bp 134 to 140 °C/1 mm, contained equal amounts of *m*-and *p*-isomer. *Fraction 5*, bp 140 to 148 °C/1 mm, was almost pure *p*-isomer. Analysis for C, H, and N indicated the same elementary composition for all fractions.

*Analysis:* Calculated for C_10_H_12_F_4_N_2_: C, 50.9; H, 5.1; N, 11.8. Found: C, 50.9; H, 5.1; N. 11.7.

Nuclear magnetic resonance analysis showed that the predominant isomer in Fraction 3 had three different fluorine-bond peaks, one twice as large as the other two, indicating the *meta* isomer; the high-boiling isomer (Fraction 5) showed only one fluorine bond and was, therefore, the *para* compound. The *meta* and *para* isomers were obtained relatively pure by vapor-phase chromatography; the *ortho* isomer was present in such small proportion as to warrant no further purification.

#### d. *p*-Bromotetrafluoroaniline

Thirty grams of bromopentafluorobenzene and 70 ml of 28-percent ammonium hydroxide were placed in a 110-ml bomb, heated to 200 °C, and shaken at this temperature for 2 hr. The product was purified as previously described for pentafluoroaniline. The yield of crystalline product, mp 61 °C, was 22 g (77%). Nuclear magnetic resonance indicated that the compound was the *para* isomer.

*Analysis:* Calculated for C_6_H_2_BrF_4_N: C, 29.5; H, 0.8; N, 5.7; Br, 32.7. Found: C, 29.4; H, 0.8; N, 5.8; Br, 32.8.

#### e. Tetrafluoro-*p*-iodoaniline

Sixteen grams of pentafluoroiodobenzene and 30 ml of 8-percent aqueous ammonium hydroxide were heated to 165 °C in a 43-ml bomb and shaken at this temperature for 2 hr. The product was purified as described previously, except that, after sublimation, the compound was recrystallized from petroleum ether, yielding 7.6 g (46.7%) of slightly yellow crystals; mp 77 °C. Nuclear magnetic-resonance spectra indicated it to be the *para* isomer.

*Analysis:* Calculated for C_6_H_2_F_4_IN: C, 24.7; H, 0.7; N, 4.8; I, 43.6. Found: C, 24.7; H, 0.8; N, 4.6; I, 42.9.

#### f. *p*-(Benzyloxy)tetrafluoroaniline

Twenty grams of benzyl pentafluorophenyl ether and a large excess of 28-percent aqueous ammonium hydroxide were heated in a 110-ml bomb to 160 °C and shaken at this temperature for 2 hr. A small amount of white crystals was observed clinging to the inside of the bomb. This material was removed and recrystallized three times from ethanol. Yield: 3 g (13%) of slightly yellow needles, mp 97 °C; it was shown by nuclear magnetic resonance to be the *para* isomer.

*Analysis:* Calculated for C_13_H_9_F_4_NO: C, 53.1; H, 3.3; N, 5.1. Found: C, 53.0; H, 3.4; N, 4.9.

#### g. 2-Chlorotrifluoro-*α*,*α,α*-trifluoro-*p*-toluidine

A mixture of 50 g of 2-chlorotetrafluoro-*α*, *α*, *α*- trifluoro-toluene and 120 ml of 28-percent ammonium hydroxide was heated to 210 °C in a 200-ml bomb and shaken at this temperature for 2 hr; the crude substituted toluidine was purified by sublimation, giving 22 g (43%) of white crystals which readily decomposed at room temperature in the presence of air (accounting for discrepancies in the analysis).

*Analysis:* Calculated for C_7_H_2_ClF_6_N: C, 33.7; H, 0.8; N, 5.6; Cl, 14.2. Found: C, 32.6; H, 1.1; N, 6.2; Cl, 14.9.

#### h. 2,3,5,6-Tetrafluoro-*p*-anisidine

To 100 ml of anhydrous liquid ammonia and 0.1 g of ferric nitrate at −70 °C was added, in small pieces, 2.99 g (0.13 g-atom) of sodium. When the blue color had permanently disappeared, 25 g (0.13 mole) of pentafluoroanisole was added slowly during 45 min. The mixture was stirred for 5 hr at −70 °C, and the ammonia was allowed to evaporate overnight at room temperature. After the addition of 100 ml of water, the fluorocarbon layer was separated, and the aqueous layer was extracted with three 50-ml portions of dichloromethane. The unreacted pentafluoroanisole, 7 g (28%), was removed by distillation; bp 137 to 138 °C. By vacuum sublimation (80 °C/1 mm) of the residue, tetrafluoro-*p*-anisidine, 2.8 g (14% based on reacted pentafluoroanisole), was obtained as white needles, mp 75 to 76.5 °C; the structure was identified by nuclear magnetic resonance.

*Analysis:* Calculated for C_7_H_5_F_4_NO: C, 43.05; H, 2.56; N, 7.19. Found: C, 43.10; H, 2.7; N, 7.1.

After removal of the above compound, the residue liquefied, and 1.2 g (6.7%) of solid 4,4′-dimethoxy-octafluorodiphenylamine was obtained by distillation, bp 80 to 82 °C/1 mm. Sublimation at 80 °C/1 mm, and recrystallization from petroleum ether gave white needles, mp 78 to 79 °C. The melting point of a mixture of the fluoroanisidine and the diphenylamine compound was depressed. Nuclear magnetic resonance showed that a substituted anisyl group was linked to the nitrogen atom at the *para* position.

*Analysis:* Calculated for C_14_H_7_F_8_NO_2_: C, 45.0; H, 1.88; N, 3.75. Found: C, 45.4; H, 2.0; N, 3.5.

The residue gave 4,4′,4″-trimethoxydodecafluorotriphenylamine as a yellow oil; 2.2 g; bp 157 to 159 °C/1 mm; $n_{D}^{23}$ 1.5005.

*Analysis:* Calculated for C_21_H_9_F_12_NO_3_: C, 46.4; H, 1.66; N, 2.57. Found C, 46.7; H, 2.1; N, 2.50.

### 5.4. Diazotization of Pentafluoroaniline

#### a. Pentafluoroiodobenzene

In a 250-ml, 3-necked, Monel flask (fitted with a metal inlet tube, Monel reflux condenser, and Teflon-covered, magnetic stirring-bar) was placed 20 g (0.108 mole) of pentafluoroaniline. Approximately 75 ml of anhydrous hydrogen fluoride was condensed into the flask at −20 °C and 7.27 g (0.105 mole) of sodium nitrite was added during 30 min. The flask was allowed to warm to −10 °C, stirring was continued for an additional 1 hr, 17.6 g (0.106 mole) of granular potassium iodide was added during 30 min, and the mixture was allowed to warm to 25 °C in 1 hr. The residual hydrogen fluoride was removed by warming the flask at 50 °C and the mixture was poured into 100 ml of water in a Monel beaker. The organic layer was separated, washed with three 50-ml portions of saturated sodium bicarbonate solution and water, and dried (sodium sulfate). On distillation, 16.5 g (50%) of pentafluoroiodobenzene, bp 77 to 79 °C/35 mm, was obtained. Analytical, vapor-phase chromatography (Viton A column at 150 °C) showed only one product, with a retention time identical with that of pentafluoroiodobenzene.

Bromopentafluorobenzene (35% yield) was prepared by the same procedure, except that 12 g of potassium bromide and 15 g of cuprous bromide was used in place of the potassium iodide.

### 5.5. Oxidation of Pentafluoroaniline

#### a. Decafluoroazoxybenzene

In a 250-ml flask were placed 10 g (0.055 mole) of pentafluoroaniline, 100 ml of glacial acetic acid, and 25 ml (0.22 mole) of 30-percent hydrogen peroxide. At 25 °C, the solution turned blue, green, dark brown, yellow, red, and finally orange within 24 hr. During an additional 24 hr, an orange liquid separated on the bottom. The reaction was stopped by dilution with 300 ml of water. The orange liquid was separated and, when chilled in ice, solidified. Yield: 4.5 g (22%). On sublimation at 80 °C/1 mm, followed by recrystallization (decolorization with charcoal) from petroleum ether, white plates of decafluoroazoxybenzene, mp 53 to 54 °C, formed. On standing, the material acquired a green tint. Confirmation of the structure was obtained from mass-spectrometer analysis, which showed the parent mass peak at 378. The ultraviolet absorption spectrum showed two maxima, one at 230 m*µ* and the other at 296 m*µ*.

#### b. Decafluoroazobenzene

A mixture of 5 g (0.013 mole) of decafluoroazoxybenzene, 15 g of zinc powder, 5 g of ammonium chloride, 10 ml of water, and 75 ml of 95-percent ethanol was stirred under reflux for 30 min. The mixture was filtered hot, and the zinc was extracted with two 50-ml portions of hot alcohol. The alcoholic filtrates were poured onto 100 g of ice, and the tan precipitate was filtered off and dried. Sublimation at 60 °C/1 mm gave 2 g (41.5%) of orange decafluoroazobenzene, mp 57 to 59 °C. Confirmation of the structure was obtained from mass-spectrometer analysis, which showed the parent mass peak at 362. The ultraviolet absorption spectrum showed one peak at 230 m*µ*, but this had two slight inflections, one at 280 m*µ* and the other 326 m*µ*. Solutions of decafluoroazoxybenzene are yellow and show one absorption peak at 370 m*µ* in the visible range, whereas the solutions of decafluoroazobenzene are orange and absorb at 465 m*µ*.

### 5.6. Reactions With Organolithium Compounds

#### a. 2,3,4,5,6-Pentafluorotoluene

Methyllithium was prepared in a 500-ml, three- necked flask equipped with a dropping funnel, magnetic stirrer, and a fitting for introducing a continuous flow of argon. Clean lithium, 4.5 g (0.61 mole) cut into small pieces, and 200 ml of sodium-dried ether were placed in the flask. A solution of 43 g of iodomethane in 50 ml of absolute ether was added dropwise to the mixture while vigorous stirring and a constant atmosphere of argon were maintained. After the reaction of the iodomethane with the lithium had begun (as evidenced by a clouding of the ether), the mixture was cooled to −10 °C with dry ice-acetone. The temperature was maintained between −10 °C and −20 °C during the entire preparation of the methyllithium.

The ether solution of methyllithium was transferred to a dropping funnel while under an argon atmosphere, and was added dropwise to a solution of 60 g of hexafluorobenzene in 250 ml of dry pentane initially at room temperature, slight refluxing from the heat of reaction being maintained. Continuous stirring under an argon atmosphere was maintained throughout the addition and for an additional 17 hr; during the final 2 hr, the solution was refluxed gently. After being cooled to room temperature, the suspension was filtered and the filtrate was washed with water and dried (sodium sulfate). The liquid was distilled through a packed column. Yield: 34 g (70%), bp 115 °C.

*Analysis:* Calculated for C_7_H_3_F_5_: C, 46.2; H, 1.6. Found: C, 46.4; H, 1.8.

#### b. Butyl-2,3,4,5,6-pentafluorobenzene

In a 250-ml, three-necked flask (fitted with a dropping funnel, stirrer, and reflux condenser, and containing a helium atmosphere) were placed 1.86 g (0.268 g-atom) of ⅛-in. lithium wire and 30 ml of anhydrous ether. To this mixture was added 5 ml of a solution of 18.3 g (0.134 mole) of 1-bromobutane in 25 ml of anhydrous ether at 25 °C. Gentle refluxing initiated the reaction, and the remainder of the solution of 1-bromobutane was added dropwise at −10 °C. After the addition was completed, the reaction mixture was stirred for an additional 1 hr at this temperature and finally allowed to warm to room temperature. The mixture was filtered, under helium, directly into a dropping funnel, and was slowly added to a solution of 25.3 g (0.134 mole) of hexafluorobenzene in 25 ml of anhydrous ether at −10 °C. The mixture was allowed to warm slowly to room temperature, refluxed for 90 min, and cooled in ice, and 100 ml of ice water added. It was then filtered and the ethereal layer was separated, dried (sodium sulfate), evaporated, and distilled. After removal of 10.5 g (30%) of unreacted hexafluorobenzene, there was obtained 7 g (33.2% based on reacted hexafluorobenzene) of butyl-2,3,4,5,6-pentafluorobenzene; bp 86 to 87 °C/25 mm; $n_{D}^{20}$ 1.4229.

*Analysis:* Calculated for C_10_H_9_F_5_: C, 53.31; H, 4.02. Found: C, 54.0; H, 4.2. There was also obtained 2.5 g (11%) of a compound, bp 230 °C/1 mm, $n_{D}^{20}$ 1.4683, which, from its chemical analysis, appeared to be impure tributyldifluorobenzene. This product was not investigated further.

*Analysis:* Calculated for C_18_H_28_F_2_: C, 76.9; H, 10.2. Found: C, 76.5; H, 11.9.

#### c. 2,3,4,5,6-Pentafluorobiphenyl

In a 1-liter, three-necked flask (equipped with a stirrer having a polytetrafluoroethylene blade, a Friedrichs condenser carrying a drying tube, and a pressure-equalizing dropping-funnel fitted with a nitrogen inlet) was placed 32.7 g (0.18 mole) of hexafluorobenzene in 150 ml of anhydrous ether. A solution of 0.18 mole of phenyllithium in 250 ml of ether was added dropwise under a nitrogen atmosphere, with constant stirring. After the first 25 ml had been added, heat was applied to initiate a vigorous exothermic reaction marked by the formation of a heavy, white precipitate. The remainder of the phenyllithium was added carefully so that the refluxing was vigorous but not violent. The mixture was stirred for 24 hr at room temperature and then treated with about 200 ml of 10 percent aqueous hydrochloric acid to destroy excess phenyllithium and to dissolve the precipitated lithium fluoride.

The aqueous layer was separated and extracted with several portions of ether. The ether solutions were combined, washed with small portions (10 ml) of saturated sodium bicarbonate solution until the washing remained basic and with saturated sodium chloride solution until neutral, and dried (sodium sulfate). Most of the ether was removed by evaporation at atmospheric pressure through a 16-in. Vigreux column; on cooling the concentrated solution, a white precipitate formed. This was removed by filtration and was shown by nuclear magnetic resonance to be 2,3,5,6-tetrafluoro-*p*-terphenyl, mp 220 °C. Yield: 8.5 g (17%).

*Analysis:* Calculated for C_18_H_10_F_4_: C, 71.5; H, 3.3; F, 25.1. Found: C, 68.3; H, 3.2; F, 24.7.

The filtrate was evaporated under Vigreux column to remove the residual ether. Sublimation of the residue gave white crystals which appeared to he impure 2,3,4,5,6-pentafluorobiphenyl. Yield: 33 g (70%); mp 69 °C.

*Analysis:* Calculated for C_12_H_5_F_5_: C, 59.0 H, 2.1; F, 38.9. Found: C, 59.5; H, 2.4; F, 37.5.

#### d. 2,3,4,5,6-Pentafluoro-*α*-methylstyrene

In a 1-liter, three-necked vessel (equipped similarly to the flask used in the addition of phenyllithium) was placed a solution of 18.6 g (0.1 mole) of hexafluorobenzene in 100 ml of anhydrous ether. A solution of isopropenyllithium in 250 ml of ether (prepared from 12.1 g of purified 2-bromopropene by the method of Braude and Evans [57]) was transferred under helium to the dropping funnel and added dropwise to the hexafluorobenzene solution. After 25 ml of reagent had been added, heat was applied to initiate refluxing; the solution became cloudy at once. On completing the addition of the isopropenyllithium, the mixture was refluxed for 1 hr and then kept overnight at room temperature. After addition of about 50 ml of 5- percent sulfuric acid, the solid was removed by filtration through a sintered-glass funnel. The ether layer of the filtrate was separated from the aqueous layer, washed with small portions of water until neutral, and dried (sodium sulfate) overnight. Fractional distillation through a 16-in. Vigreux column gave, after removal of the ether, 9 g of hexafluorobenzene and 5 g of 2,3,4,5,6-pentafluoro-*α*-methylstyrene; bp 72 to 74 °C/52 mm. Yield: 24 percent (50% based on unrecovered hexafluorobenzene).

*Analysis:* Calculated for C_9_H_5_F_5_: C, 51.9; H, 2.4; F, 45.9. Found: C, 51.8; H, 2.6; F, 44.6.

About 0.1 g of a higher-boiling liquid was isolated. Vapor-phase, chromatographic analysis (Viton A column at 125° C) of this fraction revealed the presence of two main components. The first component had the same retention time as 2,3,4,5,6-pentafluoro-*α*-methylstyrene. The second component (about 50% of the fraction) had a considerably longer retention time and may possibly have been a disubstituted derivative of hexafluorobenzene. No further attempt was made to identify the higher-boiling product.

A considerable amount of a crystalline product, not readily soluble and apparently polymeric in nature, was found in the filtered solid. Hence, it appears that anionic polymerization of the olefinic products occurs quite readily under the conditions of the reaction.

#### e. 2,3,4,5,6-Pentafluorostyrene

Using essentially the same type of apparatus and technique as previously described, a solution of vinyllithium (prepared from 0.1 mole of phenyllithium and 0.025 mole of tetravinyltin according to the method of Seyferth and Weiner [58]) in about 150 ml of ether was added dropwise to 18.6 g (0.1 mole) of hexafluorobenzene in 50 ml of anhydrous ether. The reaction at room temperature was accompanied by precipitation of a white solid and vigorous refluxing of the solution. The flask was cooled to 0 °C about halfway through the addition, to moderate the vigorous refluxing. On completion of the addition, the mixture was kept at room temperature overnight and then refluxed for 30 min. After being cooled, the mixture was filtered, and the filtrate was flash vacuum-distilled into a trap at −78 °C. Some polymeric solid was left in the flask. The distillate was fractionated through a 12-in. column packed with glass helices. After removal of the ether, about 4 g of hexafluorobenzene was recovered. The higher-boiling residue was distilled under vacuum. A considerable quantity of solid was left in the distilling flask; it may have been polymeric vinyl derivative(s) of hexafluorobenzene. The colorless, liquid distillate, bp 34 °C/25 mm, was shown to be identical with 2,3,4,5,6-pentafluoro-styrene obtained by dehydration of 2,3,4,5,6-penta-fluoro-*α*-methylbenzyl alcohol (prepared by the reaction of pentafluorophenyl Grignard reagent with acetaldehyde [12]). Vapor-phase, chromatographic analysis showed the monomer to be of high purity. Yield: 20 percent (based on unrecovered hexafluorobenzene).

### 5.7. Reaction of Hexafluorobenzene With Lithium Aluminum Hydride and Lithium Hydride

In a 300-ml, three-necked flask equipped as described previously was placed 21 g (0.11 mole) of hexafluorobenzene in 50 ml of anhydrous ether. A slurry of 3 g (0.08 mole) of lithium aluminum hydride in 200 ml of anhydrous ether was filtered into a dropping funnel, and the filtrate was added dropwise, under a slow stream of nitrogen, to the stirred, refluxing solution of hexafluorobenzene. After all of the lithium aluminum hydride solution had been added, the mixture (containing a white precipitate) was refluxed for 8 hr. Aliquots of the mixture were periodically analyzed by vapor-phase chromatography, in order to determine the conversion of hexafluorobenzene to pentafluorobenzene as a function of time. After 1 hr, an aliquot of the ethereal solution contained two components (in addition to the ether). The component having the shorter retention time was the starting material. The other component, after isolation by preparative, vapor-phase chromatography, was shown by mass-spectrometric analysis to have a parent mass of 168, corresponding to pentafluorobenzene. The conversion of hexafluorobenzene to pentafluorobenzene was estimated to be about 30 percent. After 6 hr, the conversion was about 50 percent, and, after 8 hr, 60 percent. Further refluxing did not increase the percent conversion appreciably.

The reaction mixture was cooled to −78 °C and the excess hydrides were destroyed by adding wet ether, water, and 20-percent aqueous hydrochloric acid until the mixture was acid. The ether layer was separated, combined with the ether extracts of the water layer, and dried (sodium sulfate). The ether was removed by evaporation through a 24-in. column packed with glass-helices. The higher-boiling residue was fractionated, to give 17 g of a mixture of hexafluorobenzene and pentafluorobenzene, bp 78 to 84 °C. Some of the hexafluorobenzene (about 3 g) was apparently lost during the reaction or distillation, or both. The products were separated by preparative, vapor-phase chromatography, using a column 8 ft by ⅝ in. packed with acid-washed firebrick coated with 20 percent (by weight) of silicone oil (SE 30). The final recovery of unreacted hexafluorobenzene was 8 g; the yield of pentafluorobenzene was 7.5 g (61%, based on unrecovered hexafluorobenzene). Less than 0.5 g of disubstitution product (presumably 1,2,4,5-tetrafluorobenzene) was also isolated. About 1 g of a higher-boiling, unidentified product remained in the pot.

Several other experiments were tried, using equimolar proportions of hexafluorobenzene and lithium hydride in ether. Very little, if any, pentafluorobenzene was formed, even after long periods of reflux. The same materials were sealed in a dry, evacuated bomb, which was then heated at various temperatures (80 to 200 °C) for various times (up to 24 hr). Again, no pentafluorobenzene or other product was detected by vapor-phase, chromatographic analysis [24 by 0.25-in. column, with a packing of 30 percent (by weight) of silicone oil (Dow Corning 550) on 40–60-mesh, acid-washed firebrick] of the ether solution. However, the use of lithium aluminum hydride in 10-percent molar proportion together with equimolar proportions of lithium hydride and hexafluorobenzene in ether resulted in a 25-percent conversion to pentafluorobenzene after 48 hr of reflux.

## Figures and Tables

**Table 1:** Orientational effects of *C_6_F_5_R* with nucleophiles^a^

R	H	Br	I	CH_3_	OCH_3_	OΦ	OCH_2_Φ	Φ	NO_2_	NH_2_	NHCH_3_	N(CH_3_)_2_
Base												
												
CH_3_ONa	*p*	*p*	*p*		*p*							
C_2_H_5_ONa												*p*
C_6_H_5_ONa						*p*						
KOH	*p*	*p>o*	*p*	*p*								
NaSH	*p*											
CH_3_Li				*p*								
C_4_H_9_Li		E										
C_6_H_5_Li								*p*				
NaNH_2_					*p*					S		
NH_3_	*p*	*p*	*p*		C		*p*		*o>p*	*m>p*		
CH_3_NH_2_											*p*	
(CH_3_)_2_NH												*p*>*m*>*o*
NH_2_NH_2_	*p>o>m*											
LiAlH_4_	*p*>*o*>*m*											

a *o=ortho; m=meta*; *p = para*; S = salt formation; C = cleavage of ether moiety; E = exchange (−78 °C).

## Footnotes

2 Figures in brackets indicate the literature references at the end of this paper
